# Supplementary figures and images for: Uremic pruritus and long-term morbidities in the dialysis population
Source: PLoS One. 2020 Oct 26;15(10):e0241088. doi: 10.1371/journal.pone.0241088 (PMC7588085; doi:10.1371/journal.pone.0241088)

**S1 Figure.** Prespecified subgroup analysis of heart failure hospitalization.

**
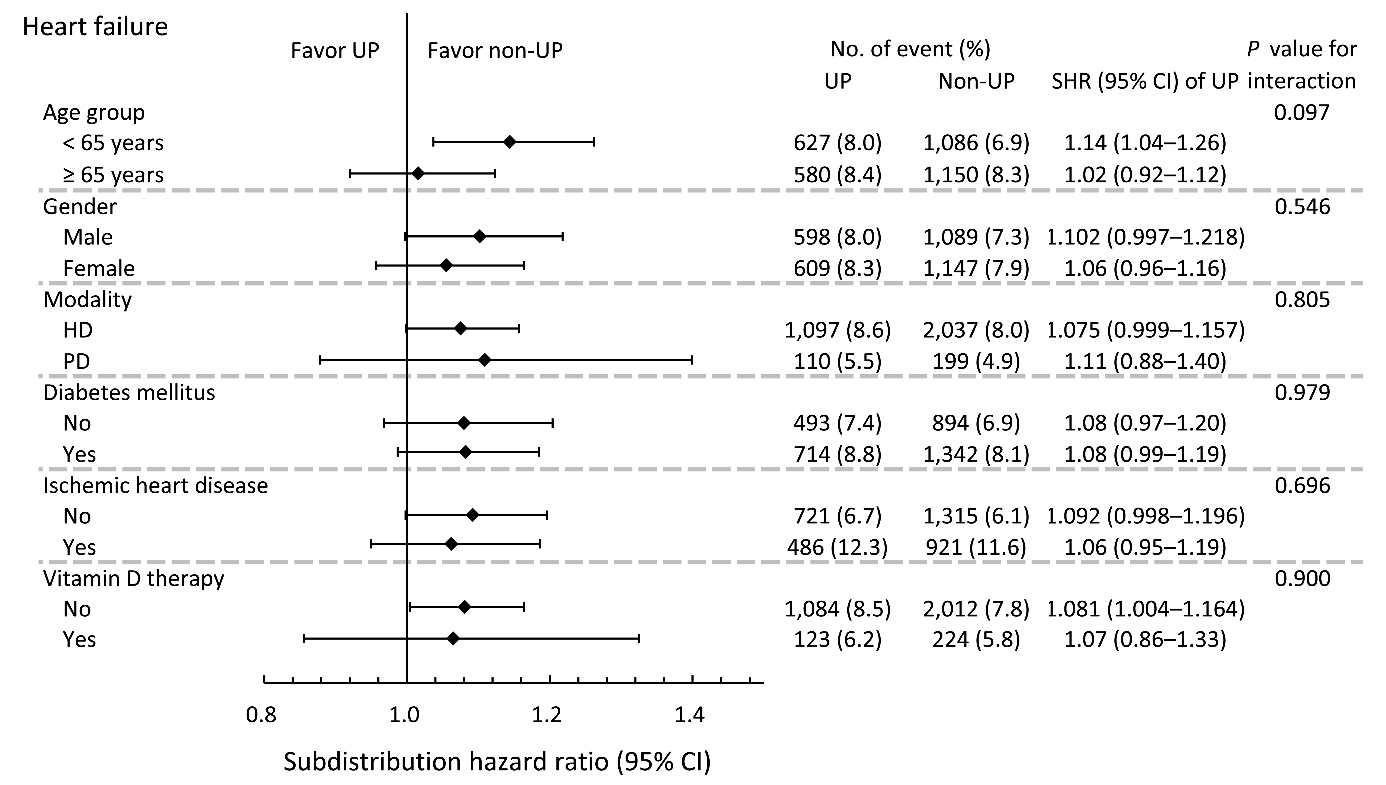
**

Supplement: S1 Fig — (DOCX) [file pone.0241088.s002.docx]

**S2 Figure.** Prespecified subgroup analysis of receiving parathyroidectomy.


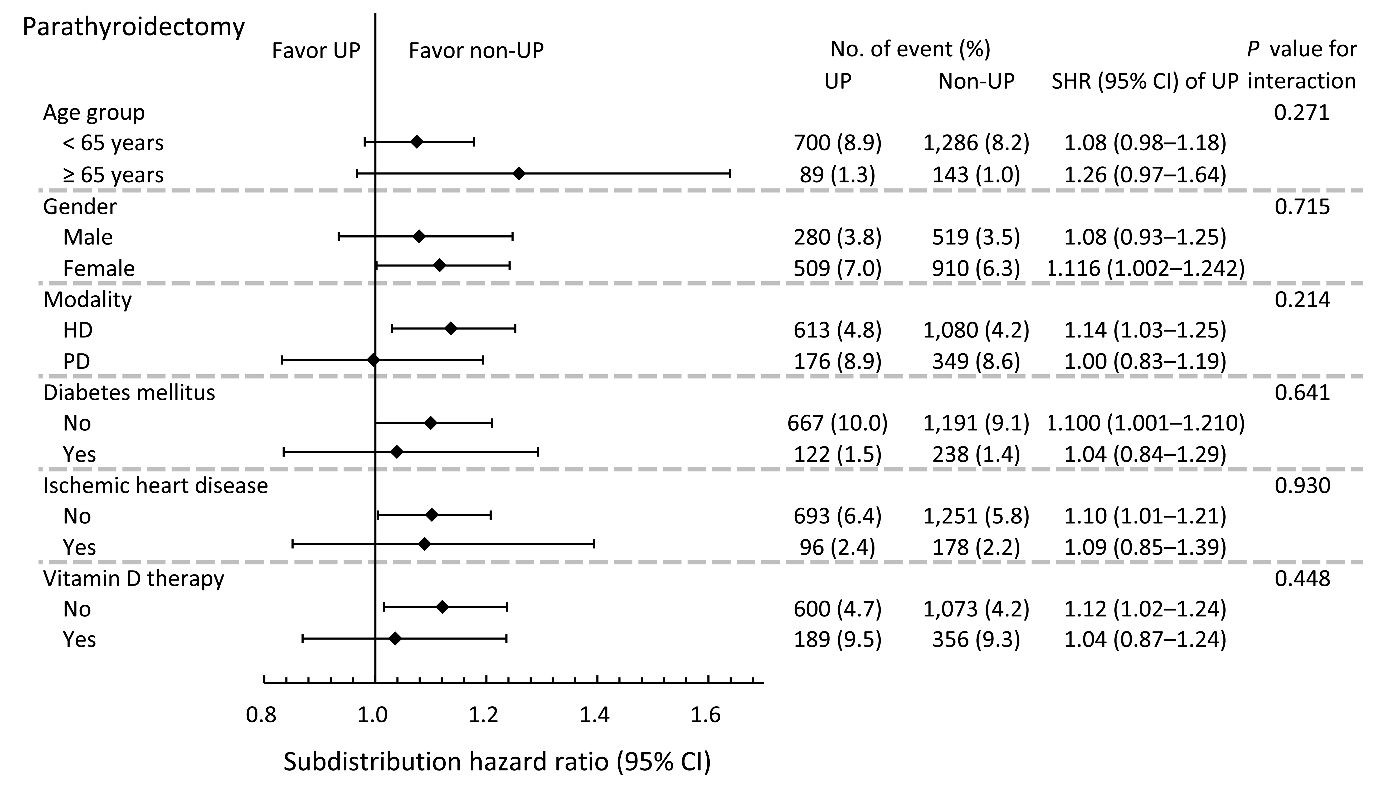

Supplement: S2 Fig — (DOCX) [file pone.0241088.s003.docx]
