# Supplementary material for: Uremic pruritus and long-term morbidities in the dialysis population
Source: PLoS One. 2020 Oct 26;15(10):e0241088. doi: 10.1371/journal.pone.0241088 (PMC7588085; doi:10.1371/journal.pone.0241088)
Supplement: Attachment — Submitted filename: PONE-D-20-17073_R1 review.pdf [file pone.0241088.s005.pdf]

Dear Authors,

All my commands have been addressed adequately.
I made the following suggestions:
1. Please include the complete information of the reference 13. Accepted, unpublished article would be cited same as published articles, but substitute “Forthcoming” for page numbers or DOI.
2. I understand the author tried to match all factors that may affect the outcomes; however, some of the factors such as the dialysis duration was not included in the factor of matching. I suggested not include the information before matching because it looked more complicated.

In general, the authors tried to revise as the commands.

Thank you very much for your attention to my opinion.
