# Supplementary material for: Uremic pruritus and long-term morbidities in the dialysis population
Source: PLoS One. 2020 Oct 26;15(10):e0241088. doi: 10.1371/journal.pone.0241088 (PMC7588085; doi:10.1371/journal.pone.0241088)
Supplement: Attachment — Submitted filename: Response to reviewers.docx [file pone.0241088.s004.docx]

**Response to reviewer 1**

Reviewer #1: In the study, the association between uremic pruritus and non-fatal long-term outcomes in dialysis patients were analyzed. The results showed that the risk of infection and cardiovascular events were increased. Here are the concerns from the reviewer:

**1. The HR was only marginally increased (HR from 1 to around 1.1) which lessened the impact of this work.**

Thanks for your comment. We agreed that a marginally increased hazard ratio may imply that uremic pruritus contribute only a small portion to the adverse outcome in dialysis patients. However, we considered this result somehow reasonable, because other factors, such as traditional cardiovascular risk factors (diabetes, hypertension, dyslipidemia) and dialysis-specific risk factors, including hyperparathyroidism, deranged calcium-phosphate balance all have their effects on the morbidities of dialysis population. Uremic pruritus can be viewed as one comorbidity which is not negligible for predicting patient outcomes.

**2. What was the reason to exclude patients with parathyroidectomy?**

Thanks for your comment. In some dialysis patients, an elevated parathyroid hormone with refractory pruritus could be the indication of parathyroidectomy, and some case series showed that parathyroidectomy may help relieve the extent of pruritus. We would like to observe this surgery during follow up, therefore, patients who underwent parathyroidectomy were excluded because they could not have this surgery again (if total parathyroidectomy) or the chance will decrease (if subtotal parathyroidectomy).

**3. For the outcomes, did the authors only count those with new events and exclude those with prior history, like what they did for parathyroidectomy?**

For cardiovascular events, the disease burden may increase over time and episodes. We counted the events after index date, but used the past history for propensity score matching. For most of the infection, we counted the events after index date without considering the past history of infection.

**4. What was the definition of infection-related hospitalization? There is a large spectrum of infection that requires hospitalization. Please try to classify the spectrum of infections, eg. viral/fungal/bacterial, common/opportunistic etc.**

We are sorry for we did not report the definition of infection-related hospitalization in the original manuscript. We used the discharged diagnoses of selected infection-related ICD-9 diagnostic codes in each emergency department visit or hospitalization. By using these ICD-9 codes, we could differentiate the sites of infection. However, the pathogen causing infection is unable to identify by using ICD-9 code. This part is addressed in the method (Definitions of Outcomes subsection) and limitation sections of revised manuscript.

**5. The definition of UP, "more than 42 daily doses of antihistamine", was a bit loose. How did the authors exclude pruritus caused by xerosis or xerotic dermatitis which is also a very common problem in dialysis patients.**

Thanks for your comment.

Xerosis is a common phenomenon in dialysis patients, and is sometimes viewed as one of the contributing/aggravating factors in uremic pruritus. If the itching improves a lot after application of emollient

We considered uremic pruritus as a “syndrome” rather a specific disease entity, therefore, we used a treatment-based criterion to select dialysis patients who has a considerable severity of itching. In previous studies, the diagnosis of uremic pruritus usually based on questionnaires or visual analogue scale, with the exclusion of primary cutaneous diseases that may require long-term use of systemic therapies, such as chronic urticaria and atopic dermatitis. The utilization of questionnaire was not diagnostic for uremic pruritus, but for classification of itching severity.

During the study period, no mu-opioid receptor antagonist of kappa-opioid receptor agonist is available for treating uremic pruritus in Taiwan. Meanwhile, the prescription of gabapentinoids are not covered by national health insurance (NHI), therefore, the first line treatment for pruritus in dialysis patients are antihistamines.

In the revised manuscript, we tried to sort out patients with more severe pruritus by increasing the threshold of antihistamine prescription to be equal or more than 84 days. Patients with any concomitant diagnoses that may require frequent antihistamine therapy are also excluded. The flowchart, all tables and figures and the text have been revised accordingly.

**6. What is the meaning of "non-fatal outcomes"? Infection and MACCE very often resulted in death, especially in immunocompromised patients. How did the authors tell "fatal" and "non-fatal" in this study?**

Thanks for your comment. First, to avoid making confusion to the readers, we would like to adjust the term “non-fatal outcomes” into “outcomes” in the revised manuscript and “long-term morbidities” in the revised title. Second, as for the outcomes in our study, we only count those did not lead to death. That is to say, we count all emergency visit or hospitalization that patients were alive at discharge. The statistical analysis also utilized subdistribution hazard model, in which death is considered a competing risk.

**Response to reviewer 2**

**1. Your previous study in reference 13 discussed the association between uremic pruritus and long-term outcomes in patients undergoing dialysis. From the big database, it would be acceptable if the data presented were in sequence of a different sample size. I would strongly suggest to review the data presented.**

Thanks for your comment. In correspondence with the other reviewer’s requirement, we changed to a stricter criterion of anti-histamine prescription to define uremic pruritus. The flowchart (and the number of eligible case), all tables and figures and the text have been revised accordingly.

**2. You used standard deviation (STD) in Table 1 to represent the difference between 2 groups. It would be more clear to use p-value to show the results.**

We thank for your comment. We used “standardized difference” (STD) because of the huge sample size. The p-value tends to be extremely low when the sample size is huge. In this situation, using the standardized difference is more suitable because it is not affected by the size of sample.

**3. The information before matching does not need to be provided in table 1. It looked more complicated and I would suggest moving these to supplement data.**

Thanks for your opinion. We prefer to demonstrate the data before and after matching in the main text, because we think it is necessary to show the real-world characteristics (data before matching) of patients with and without uremic pruritus. Showing the data after matching is also necessary because the readers can easily assess the quality of matching.

**4. Your topic is about the association of uremic pruritis and non-fatal outcomes. It seems not suitable to put fatal outcomes in Table 2 as results.**

Thanks for your reminding. We removed all the term of “fatal outcome” in this revised manuscript.

**5. In Figure 3, you did the subgroup analysis according to fatal outcomes. It would seem more suitable to do the analysis related to the non-fatal outcomes which is more related to your topic.**

We appreciate with your constructive suggestion. Based on the revised design, we performed subgroup analysis for selected morbidities/events which were significant between groups, including infection-related hospitalization, catheter related infection, major cardiovascular and cerebral composite adverse event, heart failure and parathyroidectomy. The Figure 3A-3B (forest plot for subgroup analysis) is revised and Supplemental Figure 1-3 have been added in this revised manuscript.
