# Supplementary material for: Uremic pruritus and long-term morbidities in the dialysis population
Source: PLoS One. 2020 Oct 26;15(10):e0241088. doi: 10.1371/journal.pone.0241088 (PMC7588085; doi:10.1371/journal.pone.0241088)
Supplement: Attachment — Submitted filename: Response to the reviewers.docx [file pone.0241088.s006.docx]

**Response to the reviewers**

***Reviewer #1****: The current version showed substantial improvement. Some concerns remained from the reviewer:*

*1. The study populations is still a concern although the authors made efforts to re-define the populations. Did the authors exclude those who already started using antihistamine prior to the index date in the UP group? UVB treatment is frequently applied on psoriasis and vitiligo patients. Psoriasis was excluded as UP group. How about vitiligo?*

Thanks for your comment. We agreed that the current flowchart may count patients with vitiligo who also receive UVB therapy. The patients diagnosed with vitiligo were identified with the ICD-9 code 709.01. Within 52,357 patients who have any doses of antihistamine prescription or UVB therapy, only 24 patients were found to have vitiligo (24 / 52357 = 0.05%). This low prevalence of vitiligo is compatible with Chen et al., who also used Taiwan NHIRD and found a prevalence around 0.064% in general population of Taiwan (Comorbidity profiles in association with vitiligo: a nationwide population-based study in Taiwan. Chen YT, Chen YJ, Hwang CY, et al. J Eur Acad Dermatol Venereol. 2015 Jul;29(7):1362-9. doi: 10.1111/jdv.12870.). Because of the extremely low prevalence of vitiligo in our advanced CKD & ESRD cohort, we think it may barely affect the events of our study. We have written this limitation into the limitation section.

We did not exclude patients who use antihistamine before index date, because uremic pruritus is also very frequent at the stage of advanced chronic kidney disease, thus, they may use antihistamines before they initiate dialysis. Some patients’ pruritus improves after starting dialysis therapy; however, others may continue to have itching despite the dialysis. Hence, the main interest in this study focus on the long-term impact of uremic pruritus which still exist after starting dialysis. We addressed this focus in the revised manuscript.

*2. Please recheck the panels in figure 3 and supplementary figure 1. The panels were mistaken here. Catheter-related infection should be placed in the main manuscript rather than in the supplementary data since it was a focus of discussion in the main text.*

Thanks for your suggestion. We have put supplementary fig 1 and figure 3A-B together (revised to be figure 3A-C)

*3. Supplementary Table 1: "Urticarial" should be "urticaria".*

Thank you very much. We made the correction in the revised supplemental document.

***Reviewer #2****:*

*Dear Authors,*

*All my commands have been addressed adequately.*

*I made the following suggestions:*

*1. Please include the complete information of the reference 13. Accepted, unpublished article would be cited same as published articles, but substitute “Forthcoming” for page numbers or DOI.*

Thank you very much. This research letter has been published in July 2020. We complete the information (including journal volume, issue and page) of this reference in the revised manuscript.

*2. I understand the author tried to match all factors that may affect the outcomes; however, some of the factors such as the dialysis duration was not included in the factor of matching. I suggested not include the information before matching because it looked more complicated.*

Thanks for your comment. The variables listed in Table 1 were all included in the calculation of propensity score, where the “follow up duration” indicated the duration from index date to the end of follow up. We did not include dialysis vintage in table 1, because we start the follow up at the first day that patients start dialysis; therefore, at baseline, all patients’ dialysis vintage is zero. Based on the above reason, we tend to retain data of both the before and after matching.
