# Supplementary material for: Uremic pruritus and long-term morbidities in the dialysis population
Source: PLoS One. 2020 Oct 26;15(10):e0241088. doi: 10.1371/journal.pone.0241088 (PMC7588085; doi:10.1371/journal.pone.0241088)
Supplement: Attachment — Submitted filename: Response to the reviewers_1006.docx [file pone.0241088.s007.docx]

**Response to the reviewers**

***Reviewer #1****: The questions were addressed properly. There is one minor point. The legend of figure 3 was not revised according to the change of the panels.:*

Thank you very much. We revised the figure legend of figure 3.
